# Characterization of the complete chloroplast genome of *Hordeum jubatum* (Poaceae: Pooideae: Triticeae) and phylogenetic analysis

**DOI:** 10.1080/23802359.2021.1972868

**Published:** 2021-09-15

**Authors:** Zhenjiang Chen, Yuanyuan Jin, Xiuzhang Li, Xuekai Wei, Chunjie Li, James F. White, Zhibiao Nan

**Affiliations:** a State Key Laboratory of Grassland Agro-Ecosystems, Lanzhou University, Lanzhou, China; b Key Laboratory of Grassland Livestock Industry Innovation, Ministry of Agriculture and Rural Affairs, Lanzhou University, Lanzhou, China; c Engineering Research Center of Grassland Industry, Ministry of Education, Gansu Tech Innovation Centre of Western China Grassland Industry, Lanzhou University, Lanzhou, China; d Centre for Grassland Microbiome, Lanzhou University, Lanzhou, China; e College of Pastoral Agriculture Science and Technology, Lanzhou University, Lanzhou, China; f Qinghai Academy of Animal and Veterinary Science, Qinghai University, Xining, China; g Department of Plant Biology, Rutgers University, New Brunswick, NJ, USA

**Keywords:** Wild *Hordeum jubatum*, chloroplast genome, phylogenetic analysis, perennial herb, salt and alkali

## Abstract

*Hordeum jubatum* is a salt tolerant forage, which plays an important role in improving saline-alkali land and animal husbandry alkali-saline grassland. *Hordeum jubatum* has been gradually domesticated as an ornamental grass due to its special flower color. However, no domesticated varieties of *H. jubatum* plant have been reported worldwide. This study reported the complete chloroplast genome of wild *H. jubatum*, which was 136,871 bp in length, containing a pair of inverted repeats (IRA/IRB) of 21,608 bp separated by a small single-copy (SSC) area region of 12,799 bp and the large single-copy (LSC) region of 80,856 bp. A total of 133 genes, including 85 protein-coding genes (79 PCG species), 40 transfer RNA genes (32 tRNA species), and eight ribosomal RNA genes (four rRNA species) were predicted from the chloroplast genomes. The overall GC content was 38.25%, and the corresponding values of the LSC, SSC, and IR were 36.22%, 32.15%, and 43.85%, respectively. The phylogenetic analysis showed that wild *H. jubatum* was clustered closely with *Hordeum bogdanii*.

*Hordeum jubatum* L. commonly known as foxtail barley is a perennial herb that belongs to the genus *Hordeum* of Poaceae. It has tufted, smooth and glabrous culms, up to 45 cm height; flat, rough leaves, soft green or slightly purplish spikes, short stiff cilia on the edges; and usually degenerate to awn like florets which are rarely male (Best et al. 1978). The foxtail barley is distributed in Hei Longjiang, Jilin, Liaoning, Inner Mongolia, Gansu, Shandong, northern farming pastoral ecotone, and other provinces and regions (Chao et al. 2016). It has a strong re-productive, adaptive and competitive ability to easily establish, extend and naturalize on a new habitat to develop as a kind of invasive plant. *Hordeum jubatum* has a wide range of adaptability and strong resistance to salt and alkali, and it can tolerate soil pH range from 6.4 to 9.5 (Chao et al. 2016). It can also tolerate 0.3–0.9% soil salt level and survive for several weeks under the condition of 1.5% sodium chloride (Badger and Ungar 1990; Israelsen et al. 2011). It became a dominant plant in many types of grassland, especially in saline alkali grassland (Orton 1980; Badger and Ungar 1990).

*Hordeum jubatum* begins its reproductive growth in early May in spring, exhibiting pink green and sometimes reddish spikelets (Rick 1999). Spikelets and rachises turn golden yellow when they mature (June–August), where the awns and glumes have beautiful posture (Rick 1999; Jonathan et al. 2012). Wild *H. jubatum* is often planted as an ornamental plant for landscaping and garden greening (Tong and Liang 2014). Chloroplast genome as independent units of heredity has been widely applied to understand plant molecular systematics and trace the origin of species and their migration (Clegg et al. 1994; Wolfe and Randle 2004; Cheon et al. 2017). In this study, the complete chloroplast genome sequence of wild *H. jubatum* was assembled and analyzed by NovoPlasty software (version: 3.6; parameter: k-mer = 39) (Nicolas et al. 2016).

Fresh leaves of wild *H. jubatum* were collected from grassland in Huang Chen region of Su Nan Yugu Autonomous County, southern Gansu province, China (37°54′24″N, 101°48′30″E). Genomic DNA extraction and sequencing library construction were conducted by Benagen Technology Services Limited (Wuhan, China), while qualified library (NEBNext^®^ Ultra™ II DNA Library Prep Kit for Illumina^®^) sequencing was conducted on an Illumina NovaSeq platform. Original data were filtered to get clean reads using the SOAPnuke software (version: 2.1.0). The clean reads were assembled into a complete chloroplast genome by using NovoPlasty software (version: 3.6; parameter: k-mer = 39) (Nicolas et al. 2016). Genomic alignment between the sample genome and close-related genome was performed using the BLAST tool (version: BLAST 2.9.0+; parameter: *e*-value 1e–5) (Kent 2002), and the assembled chloroplast of sample genome was annotated using GeSeq software (Michael et al. 2017). The phylogenetic tree was constructed by the maximum-likelihood method to determine the phylogenetic position of wild *H. jubatum* based on its complete chloroplast genome.

The chloroplast sequence of wild *H. jubatum* was 136,871 bp, including the large single copy region (LSC) of 80,856 bp and a small single-copy region (SSC) of 12,799 bp, and a pair of 21,608 bp inverted repeat regions (IRA/IRB). The nucleotide composition was asymmetric (30.56% A, 19.29% G, 18.96% C, and 31.18% T) with an overall GC content of 38.25%, including 36.22% in LSC region, 32.15% in SSC region, and 43.85% in IR region. It contained 133 functional genes, including 85 protein-coding genes (79 PCG species), 40 transfer RNA genes (32 tRNA species), and eight ribosomal RNA genes (four rRNA species). Among them *trnK-*UUU, *rps*16, *trnG*-UCC, *atpF*, *trnL*-UAA, *trnI*-GAU, *trnV*-UAC, *trnA*-UGC, *rpl2*, *ndhA*, *ndhB*, *rps*12, *trnI*-GAU, *trnA*-UGC, *rps*12, *ndhB*, *rp1*2 contain a single intron, and *ycf*3 contains two introns. Most gene species occurred in a single copy. However, 19 gene species occurred in double copies, including seven PCG genes (*rps*7, *rps*12, *rps*15, *rps*19, *rpl*2, *rpl*23, and n*dhB*), eight tRNA genes (*trnN*-GUU, *trnR*-ACG, *trnA*-UGC, *trnl*-GAU, *trnV*-GAC, *trnL*-CAA, *trnl*-CAU, *trnH*-GUG), and four rRNA genes (*rrn*4.5, *rrn*5, *rrn*16, and *rrn*23).

The phylogenetic relationships were determined with the complete chloroplast genome of wild *H. jubatum* and other 23 grass species (Poaceae) available from the National Center of Biotechnology Information (NCBI) database. Phylogenetic analysis showed that the 24 species were clustered into two main clades, with *H. bogdanii* close relative to wild *H. jubatum* (Figure 1). This complete chloroplast genome of wild *H. jubatum* provides relevant data to allow a robust phylogenetic analysis within the polyploidy genus *Hordeum.*

**Figure 1.**
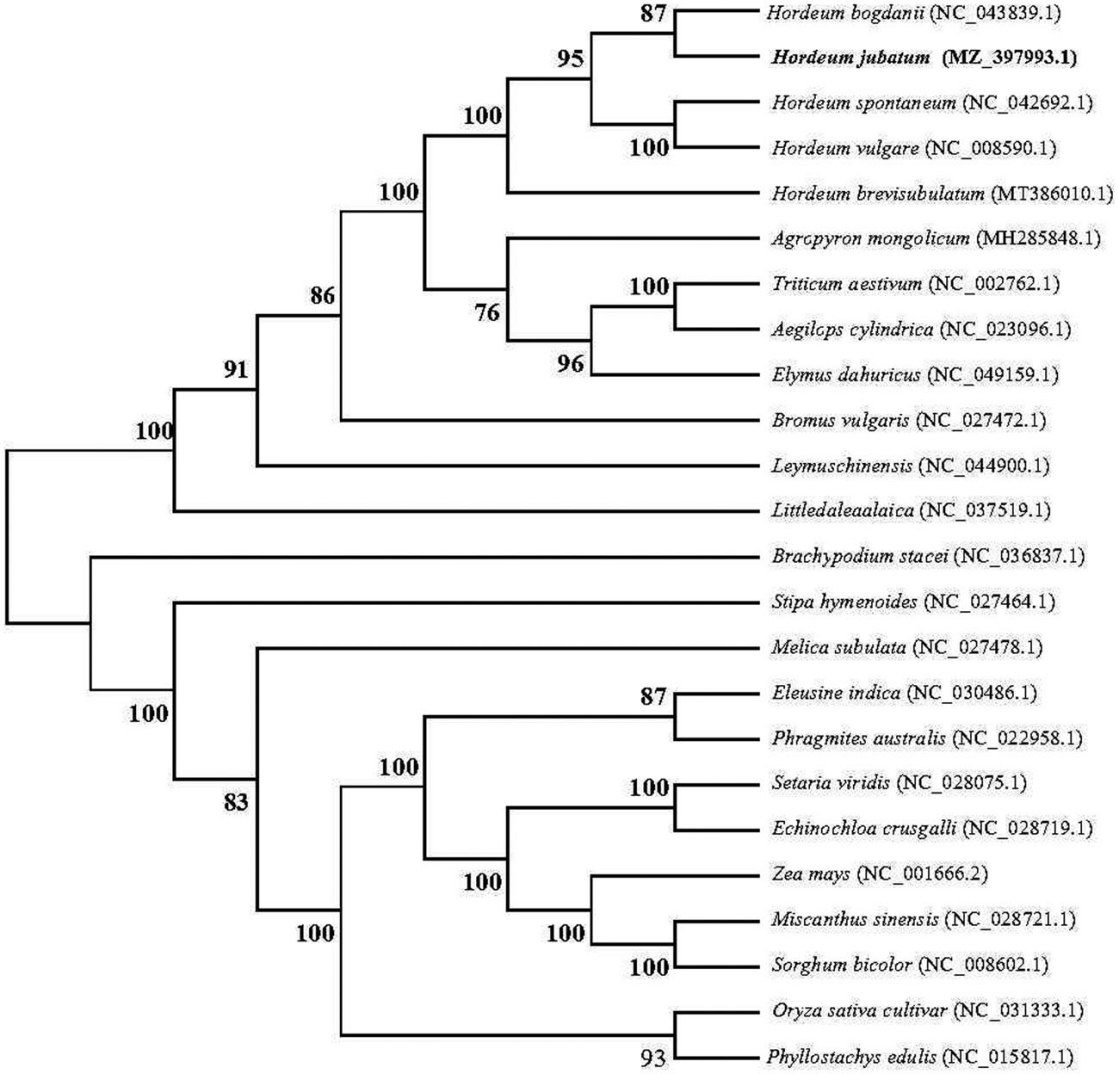
Maximum-likelihood tree of *Hordeum jubatum* plus 23 other grass species (Poaceae) based on their chloroplast genome sequences. Bootstrap support values (based on 1000 replicates) are shown next to the nodes.

## Data Availability

A plant and DNA specimens were deposited at State Key Laboratory of Grassland Agro-ecosystems, Lanzhou University, Lanzhou, China (http://sklgae.lzu.edu.cn/index.jsp; contact person: Zhenjiang, Chen, and email: chenzj@lzu.edu.cn) under the voucher number SKLGAE-SN-PHJ0107 and SKLGAE-SN-DHJ0107. The *Hordeum jubatum* data have been stored in nucleotide data-base of National Center of Biotechnology Information. GenBank accession number is MZ397993 (https://www.ncbi.nlm.nih.gov/nuccore/MZ397993). Associated BioProject is PRJNA738268 (https://www.ncbi.nlm.nih.gov/biopro-ject/PRJNA738268). BioSample accession number at https://www.ncbi.nlm.nih.gov/biosample/SAMN19717117 and Sequence Read Archive at https://www.ncbi.nlm.nih.gov/sra/SRR14826256.
